# *N*-Acetyltransferase 1 Knockout Elevates Acetyl Coenzyme A Levels and Reduces Anchorage-Independent Growth in Human Breast Cancer Cell Lines

**DOI:** 10.1155/2019/3860426

**Published:** 2019-08-20

**Authors:** Marcus W. Stepp, Raúl A. Salazar-González, Kyung U. Hong, Mark A. Doll, David W. Hein

**Affiliations:** Department of Pharmacology & Toxicology and James Graham Brown Cancer Center, University of Louisville, Louisville, KY 40202, USA

## Abstract

Elevated expression of *N*-acetyltransferase 1 (NAT1) is associated with invasive and lobular breast carcinomas as well as with bone metastasis following an epithelial-to-mesenchymal transition. We investigated the effect of NAT1 gene deletion in three different human breast cancer cell lines, MDA-MB-231, MCF-7, and ZR-75-1. Human NAT1 was knocked out using CRISPR/Cas9 technology and two different guide RNAs. None of the NAT1 knockout (KO) cell lines exhibited detectable NAT1 activity when measured using its selective substrate *p*-aminobenzoic acid (PABA). Endogenous acetyl coenzyme A levels (cofactor for acetylation pathways) in NAT1 KO cell lines were significantly elevated in the MDA-MB-231 (*p* < 0.001) and MCF-7 (*p*=0.0127) but not the ZR-75-1 (*p* > 0.05). Although the effects of NAT1 KO on cell-doubling time were inconsistent across the three breast cancer cell lines, the ability of the NAT1 KO cell lines to form anchorage-independent colonies in soft agar was dramatically and consistently reduced in each of the breast cancer cell lines. The NAT1 KO clones for MDA-MB-231, MCF-7, and ZR-75-1 had a reduction greater than 20-, 6-, and 7- folds in anchorage-independent cell growth, respectively, compared to their parental cell lines (*p* < 0.0001, *p* < 0.0001, and *p* < 0.05, respectively). The results indicate that NAT1 may be an important regulator of cellular acetyl coenzyme A levels and strongly suggest that elevated NAT1 expression in breast cancers contribute to their anchorage-independent growth properties and ultimately metastatic potential.

## 1. Introduction

Human arylamine *N*-acetyltransferase 1 (NAT1) catalyzes the transfer of an acetyl group from acetyl coenzyme A (AcCoA) to arylamine and hydrazine substrates [1, 2]. Human NAT1 also catalyzes hydrolysis of AcCoA in the presence of folate [3, 4]. NAT1 has a ubiquitous expression regulated by multiple mechanisms [5]. Elevated NAT1 expression is associated with invasive and lobular breast carcinomas [6]. Additional studies have reported elevated NAT1 expression in estrogen receptor-positive tumors [7–9] as well as with bone metastasis following an epithelial-to-mesenchymal transition (EMT) [10, 11].

A recent report demonstrated that congenic rats expressing high levels of rat *N*-acetyltransferase 2 (NAT2; ortholog to human NAT1) activity exhibited more mammary tumors, and this finding was independent of carcinogen metabolism [12]. The effects of inhibition or overexpression of human NAT1 has been the focus of previous studies [13–17]. Interestingly, MDA-MB-231 cells with increased NAT1 activity showed lower endogenous AcCoA levels, compared to the parental cell line [17]. These observations, together with the wide-spread tissue distribution of NAT1 and its presence in almost all species [18] and its ability to catalyze the hydrolysis of AcCoA, suggest the role of NAT1 in carcinogenesis might be related to the regulation of AcCoA.

In the present study, we utilized CRISPR/Cas9 to investigate the effects of NAT1 knockout (KO) on endogenous AcCoA levels and the cell growth properties in three human breast cancer cell lines that originate from separate pleural effusions of different malignant breast cancer patients frequently used in breast cancer research.

## 2. Materials and Methods

### 2.1. Construction of NAT1 KO Cell Lines

MDA-MB-231, MCF-7, and ZR-75-1 breast cancer cell lines were obtained from ATCC (Manassas, Virginia, USA). MDA-MB-231 is estrogen receptor-negative, progesterone receptor-negative, and HER2-negative. MCF-7 is estrogen receptor-positive, progesterone receptor-positive, and HER2-negative. ZR-75-1 is estrogen receptor-positive, progesterone receptor-positive, and HER2-positive. A MDA-MB-231 breast cancer cell line with a single FRT site (Life Technologies, Grand Island, NY) and a nonspecific scrambled shRNA inserted in the FRT site was used as the parent MDA-MB-231 cell line. The construction of this cell line was described previously [16]. MDA-MB-231 and MCF-7 cells were cultured in DMEM media, high glucose (4.5 g/L) with the addition of fetal bovine serum (10%), glutamine (2 mM), and Pen/Strep (1%). ZR-75-1 cells were cultured in RPMI-1640 with the addition of fetal bovine serum (10%), glutamine (2 mM), and Pen/Strep (1%). The cell lines were grown in a humidified incubator set at 37°C with 5% CO_2_. Horizon Discovery Group (Cambridge, UK) designed 5 different gRNAs for NAT1, and DNA 2.0 Inc (Menio Park, CA, USA) cloned the gRNAs into a Cas9 expressing vector expressing a dasher-GFP tag. Initially, each of the 5 gRNA/Cas9 vectors was transiently transfected in each cell line using the Amaxa Nucleofector II (Lonza, Allendale, NJ, USA). After forty-eight hours, transfection cells were harvested and DNA isolated. The SURVEYOR Mutation Detection Kit (Transgenomics, Omaha, NE, USA) was used to determine the effectiveness of each gRNA's ability to induce DNA strand breaks effectively. The gRNAs #2 and #5 were the most effective at inducing DNA strand breaks and were chosen to separately KO NAT1 as described below. The selected gRNA sequences were the following: gRNA #2, **CCA ***GATCCGAGCTGTTCCCTTTG* (protospacer adjacent motif is shown in bold face font; positions 93–112 from start shown in italic font) or gRNA #5, GAAAGAATTGGCTATAAGAAGTCT**AGG** (protospacer adjacent motif is shown in bold face font; positions 26–45 from start shown in italic font).

The parent MDA-MB-231 cell line described above was transfected with either #2 or #5 gRNA/Cas9 vectors separately as above and 48 hr after transfection cells were sorted for GFP fluorescence (MoFlo XDP, Beckman Coulter Inc. Kendall, FL, USA). MCF-7 and ZR-75-1 cells were transfected with #2 or #5 gRNA/Cas9 separately with Lipofectamine 3000 (Invitrogen, CA, USA), and 48 hr after transfection cells were sorted for GFP fluorescence as previously described. The GFP-positive cells were collected and plated at a low cell density so that individual unique clones could be isolated. After several weeks, individual cells grew into large enough colonies to utilize cloning cylinders to trypsin cells off the plate and transfer to a 96-well culture plate. Approximately 25 to 50 separate clones, chosen at random, for each cell gRNA, were passaged until nearly confluent in a 6-well plate and then were tested for PABA NAT1 activity. GFP-positive clones with undetectable PABA NAT1 activity were selected for further characterization. The NAT1 open reading frame was sequenced. We chose transient transfection of the gRNA/Cas9 protein to minimize off-target effects; thus; the gRNA/Cas9 plasmid was only present in the cell for a short time (48–96 hr) as opposed to stable long-term expression of gRNA/Cas9 where the editing machinery would be present indefinitely.

### 2.2. Sequencing of the NAT1 Gene in the gRNAs #2 and #5 KO Clones

Genomic DNA was isolated from MDA-MB-231, MCF-7, and ZR-75-1 NAT1 KO cell lines. The NAT1 open reading frame was amplified by PCR and cloned into pcDNA™3.1/V5-His-TOPO® (Invitrogen, CA, USA) following manufacturer's recommendations. TOPO cloning reaction for the individual cell lines was transformed into One Shot TOP10 chemically competent *E. coli*. For each NAT1 KO cell line, five transformed *E. coli* colonies were selected and grown overnight. Cultures of bacteria were then harvested for plasmid purification. Purified plasmids and primers were sent for DNA sequencing (Eurofins, Louisville, KY, USA) to determine base changes caused by gRNA/Cas9.

### 2.3. Cell Line Authentication

The genetically engineered MDA-MB-231 MCF-7 and ZR-75-1 cell lines described above were authenticated by the ATCC Short Tandem Repeat (STR) profiling authentication service.

### 2.4. *In Vitro and In Situ N*-Acetylation

*In vitro N*-acetylation assays using the NAT1-selective substrate PABA were conducted, and *N*-acetyl-PABA was separated and quantitated by high-performance liquid chromatography (HPLC) as previously described [16]. Briefly, enzymatic reactions containing 50 *μ*L suitably diluted cell lysate, PABA (300 *μ*M), and AcCoA (1 mM) were incubated at 37°C for 10 min. Three independent measurements (*n* = 3) performed in triplicate were completed for each cell line. In vitro *N*-acetylation assays using the NAT2-selective substrate sulfamethazine enzymatic assays were conducted as described previously [19]. Briefly, reactions containing lysate from parental and NAT1 KO cells lines for all cell lines, 300 *μ*M sulfamethazine, and 1 mM AcCoA were incubated at 37°C for 120 min. Reactions were terminated by the addition of 1/10 volume of 1 M acetic acid. The reaction tubes were centrifuged to remove precipitated protein. Sulfamethazine and *N*-acetyl-sulfamethazine were separated and quantified by reverse-phase HPLC. Three independent measurements (*n* = 3) performed in triplicate were completed for each cell line. Under the conditions of this assay, the limit of detection was 0.005 nmoles/min/mg protein.

Measurement of NAT1-catalyzed *N*-acetylation *in situ* was determined by spiking media with a known concentration of PABA as previously described [20]. Briefly, the cells were incubated at 37°C for 48 hr with media containing 500 *μ*M PABA. *N*-acetyl-PABA was separated and quantitated by HPLC as previously described [16]. The number of separate determinations for MDA-MB-231, MCF-7, and ZR-75-1 was 3, 4, and 4, respectively.

### 2.5. NAT1 and NAT2 In-Cell Western Staining

Cells (1.5 × 10^5^) were plated into 96-well black/clear bottom plates (Thermo Fisher Scientific, Waltham, MA, USA) and incubated overnight at 37°C and 5% CO_2_. Once attached to the plate, cells were washed with PBS and then fixed to the plate with 3.7% formaldehyde in PBS for 20 min at room temperature. After fixing, cells were permeabilized using 0.1% Triton-X100 in PBS for 5 min with constant agitation, and the process was repeated 4 times. Cells were blocked with Odyssey® Blocking Buffer in PBS (LI-COR Biosciences, Lincoln, NE, USA) for 1.5 hours with constant agitation. After blocking, cells were incubated with rabbit anti-NAT1 (ab109114 (1:200), Abcam, Cambridge, UK) or rabbit NAT2 (ab194114 (1:100) Abcam) and *β*-actin (A2228 (1:200), Sigma-Aldrich, St. Louis, MO, USA) overnight at 4°C with constant agitation. Due to the high similarity between human NAT1 and NAT2, we evaluated the specificity of the primary antibodies against human NAT1 and NAT2. The specificity of ab109114 was about 4-fold greater for human NAT1 than NAT2, and the specificity of ab194114 was about 7-fold greater for human NAT2 than NAT1 (manuscript in preparation). After primary antibodies incubation, plates were washed 5 times with 0.1% Tween 20 in PBS for 5 min. Secondary detection was carried out using IRDye® 800CW Goat anti-Rabbit IgG (1:1200) or IRDye® 680RD Goat anti-Mouse IgG (1:1200), (LI-COR Biosciences, Lincoln, NE, USA) by incubation for 60 min. Finally, cells were washed with 0.1% Tween 20 in PBS. NAT1 or NAT2 and *β*-actin were simultaneously visualized using an Odyssey infrared imaging Scanner (LI-COR Biosciences) using the 680 nm channel and 800 nm channel. Relative fluorescence units (RFUs) allowed a quantitative analysis. Relative protein expression was calculated by dividing RFU for NAT1 or NAT2 (800 nm channel) by the RFU of *β*-actin (680 nm channel). Protein expression in NAT1 KO cells was divided by the protein expression in the parental cell line to determine fold change. The data was generated from 4 independent measurements for MDA-MB-231, MCF-7, and ZR-75-1 parental and their NAT1 KO cell lines.

### 2.6. Endogenous AcCoA Levels

Endogenous AcCoA levels within MDA-MB-231, MCF-7, and ZR-75-1 parental and NAT1 KO cell lines were measured by HPLC as previously described [16] with minor modifications. MDA-MB-231, MCF-7, and ZR-75-1 cell lines were plated in triplicate at a density of 1 × 10^6^ cells per 10 cm plate and allowed to grow. After seventy-two hr, plating cells were washed once with 1X PBS and dissociated from the plate with 1.0 mL trypsin. Cells from 3, 10 cm plates were combined, resuspended in complete media, and counted. In the subsequent steps all cells and lysates were kept on ice. Collected cells were washed once in ice-cold PBS and transferred to a 1.5 ml microcentrifuge tubes. The suspended cells were collected by centrifugation and the supernatants discarded. Having removed any residual PBS, the cells were completely resuspended in 50 *μ*L of ice-cold 1X PBS and then immediately lysed by addition of 50 *μ*L of cold 10% 5-sulfosalicylic acid with vortexing for 15 sec. Lysed cells were incubated on ice for 10 min before centrifugation at 13,000 ×g for 10 min. Supernatant was injected on a C18 reverse-phase HPLC column (250 mm × 4 mm; 5 *μ*m pore size) (Merck, Darmstadt, GER). HPLC separation and quantitation of AcCoA were achieved as previously described [16]. The data was generated from 8, 12, and 3 independent measurements for MDA-MB-231, MCF-7, and ZR-75-1 parental and NAT1 KO cell lines, respectively.

### 2.7. Cell Doubling Time

Doubling time for each parental and NAT1 KO cell line was determined by plating each cell line to a confluence level that would give the cell lines ample room to grow for at least 7 days or 168 hr. The same number of cells were plated for parental and NAT1 KO cell lines for each cell line. Cells were plated in 6-well plates in triplicate and allowed to grow for 7 days (168 hr). Cells were counted and doubling time calculated using the online calculator (http://www.doubling-time.com/compute.php). The number of separate doubling time determinations for MDA-MB-231, MCF-7, and ZR-75-1 cells was 3, 3, and 4, respectively.

### 2.8. Anchorage-*Dependent* and Anchorage-*Independent* Growth Assays

Anchorage-*dependent* growth assays were performed as described previously [16]. Briefly, cells (300 cells/well) were plated in triplicate in 6-well plates and allowed to grow for 2 weeks. Visible colonies were counted manually following staining with crystal violet. The data were generated from 6, 3, and 3 independent measurements for MDA-MB-231, MCF-7, and ZR-75-1 parental and NAT1 KO cell lines, respectively. The anchorage-*independent* growth assays were performed as described previously [16]. Briefly, the anchorage-independent growth assays were performed by plating the cells (6000 cells/well) in 1.5 mL of low-melting temperature agarose (0.3%) in complete media over a base layer of 1.5 mL noble agar (0.5%) in complete media. The total volume was 3 mL in each well of a 6-well plate. Cells were plated in triplicate and grown for 2 weeks. Colonies (containing >4 individual cells) were counted manually following staining with crystal violet. The data was generated from 3 independent measurements for MDA-MB-231, MCF-7, and ZR-75-1 parental and NAT1 KO cell lines.

### 2.9. Statistical Analyses

Differences between the MDA-MB-231 and MCF-7 parental and NAT1 KO cell lines were analyzed for significance by ANOVA followed by Bonferroni post hoc test. Differences between the ZR-75-1 parental and NAT1 KO cell lines were analyzed for significance by Student's *t*-test. All statistical analyses were performed using GraphPad Prism v6.0c (GraphPad Software, La Jolla, CA, USA). The results are expressed as the mean ± the standard error of the mean (SEM). Values of *p* < 0.05 were considered statistically significant.

## 3. Results

### 3.1. NAT1 Genomic and Amino Acid Sequences

Sequencing the NAT1 gene of MDA-MB-231 gRNA #2 (clone 2–19) KO cell line revealed a deletion of a single cytosine at 96 bases (bp) from the translation start codon (Table 1). This single-nucleotide deletion resulted in a frameshift mutation causing a premature stop codon after amino acid 49 of 290 (Table 2). The MDA-MB-231 gRNA #5 (clone 5–50) KO cell line had two nucleotides deleted at 43 and 44 bp from the translation start codon (Table 1). This deletion resulted in a premature stop codon after amino acid codon 14 of 290, which immediately terminates translation of NAT1 (Table 2).

Sequencing the NAT1 gene of MCF-7 gRNA #2 (clone 2–4) KO cell line showed a 34 bp deletion in the open reading frame, which spans from 95 to 129 bp (Table 1). This deleted segment of DNA resulted in a frameshift mutation causing a premature stop codon after 38 amino acids (Table 2). The MCF-7 gRNA #5 (clone 5–20) KO cell line had two different deletions (Table 1). The first deletion was a single nucleotide deletion at 42 bp, and the other was a deletion of 43 to 48 bp with an additional adenosine insertion in the same region. These deletions and insertions resulted in a premature stop codon after amino acid codon 23 for both sequences (Table 2).

Sequencing the NAT1 gene of ZR-75-1 gRNA #2 (clone 2–10) KO cell line showed a single adenosine insertion at 95 bp in the open reading frame (Table 1). This insertion results in a frameshift mutation causing a premature stop codon after 37 amino acids (Table 2).

### 3.2. *In Vitro* and *In Situ* PABA *N*-Acetylation

**Figure 1:**
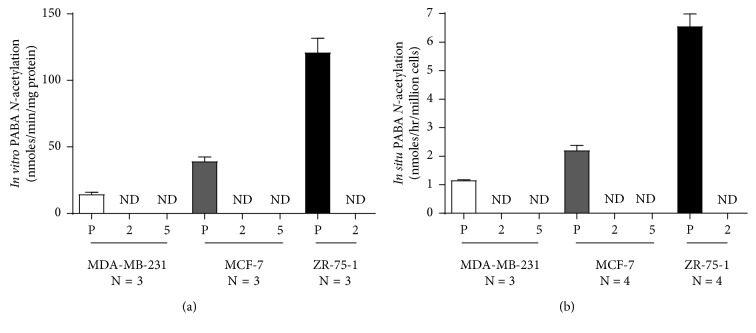
*In vitro* and *in situ* PABA (N)-acetylation activity of parental and NAT1 KO clones for MDA-MB-231, MCF-7, and ZR-75-1 cell lines. (a) The in vitro PABA N-acetyltransferase activity in MDA-MB-231, MCF-7, and ZR-75-1 parental (P) and gRNA #2 (2) and #5 (5) clones NAT1 KO cell lines are shown. (b) The *in situ* PABA *N*-acetylation in MDA-MB-231, MCF-7, and ZR-75-1 parental (P) and gRNA #2 (2) and #5 (5) clones NAT1 KO cell lines are shown. Each bar illustrates mean ± SEM. Three or four separate determinations were performed in triplicate. ND is nondetectable ((a) <0.05 nmoles/min/mg; (b) <0.20 nmoles/hr/million cells).

The *in vitro N*-acetylation of PABA in the parental cell line was 14.4 ± 2.8, 39.0 ± 5.9, and 121 ± 19 nmoles/min/mg for MDA-MB-231, MCF-7, and ZR-75-1 cell lines, respectively (Figure 1(a)). The gRNA #2 and #5 clones for all cell lines reduced levels of activity to below the limit of detection (0.05 nmoles/min/mg; Figure 1(a)). The *N*-acetylation of PABA *in situ* followed the same pattern as that for *in vitro* activity. *N*-acetylation activity of PABA in the parental cell lines was 1.13 ± 0.01, 2.20 ± 0.35, and 6.56 ± 0.87 nmoles/hr/million cells for MDA-MB-231, MCF-7, and ZR-75-1 cell lines, respectively (Figure 1(b)). In the gRNA #2 and #5 clones, levels of PABA *N*-acetylation *in situ* were reduced to below the limit of detection (0.20 nmoles/hr/million cells Figure 1(b)).

### 3.3. Human NAT1 and NAT2 Protein Levels

**Figure 2:**
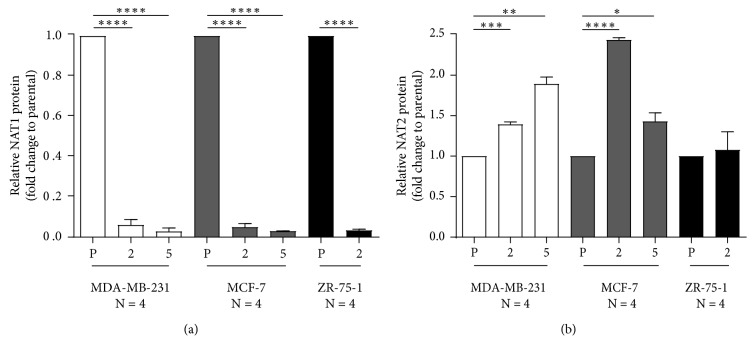
NAT1 and NAT2 protein expression in breast cancer cell lines. (a). Relative NAT1 protein expression was evaluated following an in-cell western staining protocol as described in Materials and Methods. NAT1 protein expression was significantly (*p* < 0.0001) decreased in MDA-MB-231 gRNA #2 (2) and gRNA #5 (5) NAT1 KO cells compared to the parental (P); MCF-7 gRNA #2 (2) and gRNA #5 (5) and ZR-75-1 gRNA #2 (2) NAT1 KO cells compared to the respective parental (P) cells. (b). Relative NAT2 protein expression in MDA-MB-231 gRNA #2 (2) and gRNA #5 (5) KO cells was significantly (*p* < 0.0001) increased compared to the parental (P) cells, the same phenomenon was observed for MCF-7 gRNA #2 (2) and gRNA #5 (5) KO cells (*p* < 0.0001) compared to the parental (P); however, no significant (*p* > 0.05) changes in the relative NAT2 protein expression were observed in ZR-75-1 gRNA #2 (2) NAT1 KO cells compared to the parental (P). Data expressed as mean ± SEM for 4-different determinations ^*∗*^*p* < 0.05, ^*∗∗*^*p* < 0.01, ^*∗∗∗*^*p* < 0.001, ^*∗∗∗∗*^*p* < 0.0001.

Relative NAT1 and NAT2 protein expression was evaluated following an in-cell western staining protocol as described in Materials and Methods. NAT1 protein expression was significantly (ANOVA, *p* < 0.0001) decreased in the MDA-MB-231 gRNA #2 and gRNA #5, the MCF-7 gRNA #2 and gRNA #5, and the ZR-75-1 gRNA #2 NAT1 KO cells compared to their respective parental cells (Figure 2(a)). Relative NAT2 protein expression in MDA-MB-231 gRNA #2 and gRNA #5 and in MCF-7 gRNA #2 and gRNA #5 NAT1 KO cells were increased significantly (ANOVA, *p* < 0.0001) compared to their respective parental cell line whereas no significant changes (*p* > 0.05) in NAT2 protein expression were observed in ZR-75-1 gRNA #2 NAT1 KO cells compared to the parental (Figure 2(b)). Following detection of increased NAT2 protein, sulfamethazine NAT2 enzymatic assays were conducted as described in Materials and Methods, but NAT2 activity was below the limit of detection (0.005 nmoles/min/mg protein) in all cell lines tested.

### 3.4. Endogenous AcCoA Levels

**Figure 3:**
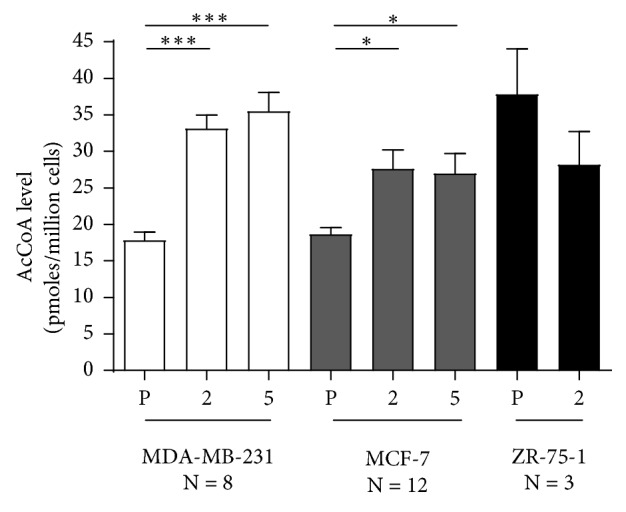
Intracellular AcCoA levels in parental and NAT1 KO clones for MDA-MB-231, MCF-7, and ZR-75-1 cell lines. AcCoA levels were measured in parental (P) and NAT1 KO cell lines for MDA-MB-231, MCF-7, and ZR-75-1. Each bar illustrates mean ± SEM for number of replicates (*N*). AcCoA levels differed significantly between parental and NAT1 KO cells for MDA-MB-231 (*p* < 0.0001) and MCF-7 (*p* < 0.05) cell lines.

The endogenous level of AcCoA within the MDA-MB-231 parental cell line was 17.8 ± 1.1 pmoles/million cells, whereas the endogenous level of AcCoA within the cells of the gRNA #2 and #5 NAT1 KO clones was 33.1 ± 1.8 and 35.5 ± 2.6 pmoles/million cells, respectively, both of which were significantly elevated compared to the MDA-MB-231 parental cell line (*n* = 8; ANOVA, *p* < 0.0001) (Figure 3).

The MCF-7 parental cell line had an endogenous AcCoA level of 18.7 ± 0.9 pmoles/million cells, whereas the endogenous levels of AcCoA within the cells of the gRNA #2 and #5 NAT1 KO clones were 27.6 ± 2.6 and 27.0 ± 2.7 pmoles/million cells, respectively, both of which were significantly elevated compared to their MCF-7 parental cell line (*n* = 12; ANOVA, *p* < 0.05) (Figure 3).

The ZR-75-1 parental cell line had an endogenous AcCoA level of 43.2 ± 3.6 pmoles/million cells, whereas the endogenous levels of AcCoA within the cells of the gRNA #2 NAT1 KO was 33.6 ± 8.4 pmoles/million cells (*n* = 3; Student's *t*-test, *p* > 0.05) (Figure 3).

### 3.5. Cell Doubling Time

**Figure 4:**
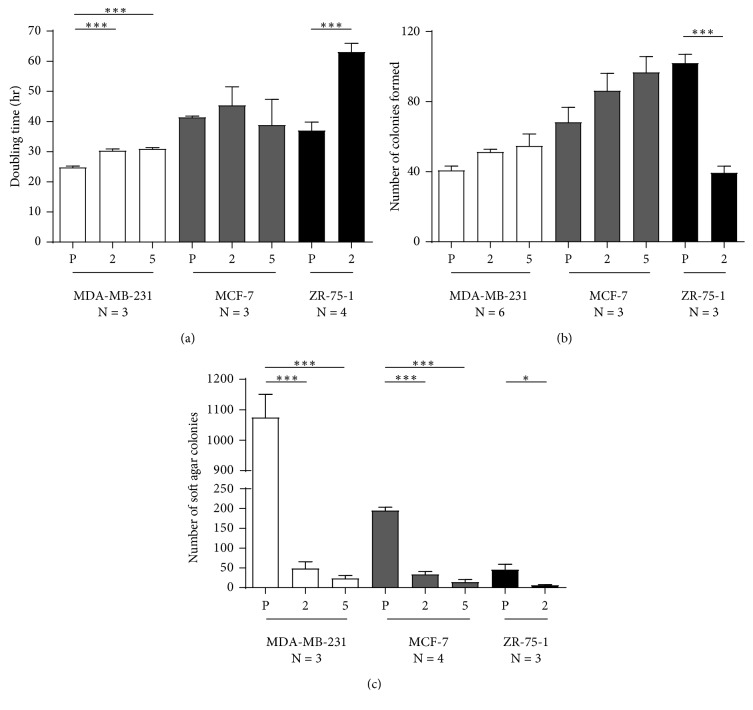
Doubling time and anchorage-dependent and anchorage-independent colony formation of parental and NAT1 KO clones for MDA-MB-231, MCF-7, and ZR-75-1 cell lines. (a) Doubling time was determined in MDA-MB-231, MCF-7, and ZR-75-1 parental (P) and gRNA #2 (2) and #5 (5) NAT1 KO clones. Each bar illustrates mean ± SEM for number of replicates (*N*). Doubling times differed significantly between parental and NAT1 KO cell lines for MDA-MB-231 (*p* < 0.0001), and ZR-75-1 (*p*=0.0006) cell lines. (b) Anchorage-*dependent* growth/colony formation was determined in MDA-MB-231, MCF-7, and ZR-75-1 parental (P) and NAT1 KO cell lines. Cells (300) were plated on plastic in triplicate and allowed to grow for 14 days before staining. Anchorage-*dependent* growth/colony formation between parental and NAT1 KO cells were not significantly different (*p* > 0.05) for all cell lines, except for ZR-75-1. (c) Anchorage-*independent*/soft agar assays were completed in MDA-MB-231, MCF-7, and ZR-75-1 parental (P) and NAT1 KO cell lines. Cells (6000) plated in triplicate in soft agar were allowed to grow for 14 days before staining. The number of colonies formed in soft agar were significantly higher in parental MDA-MB-231 (*p* < 0.0001) MCF-7 (*p* < 0.0001) and ZR-75-1 (*p* < 0.05) than their respective NAT1 KO cell lines.

The doubling times for the MDA-MB-231 parental and gRNA #2 and #5 NAT1 KO cell lines were 24.8 ± 0.3, 30.3 ± 0.4, and 30.9 ± 0.3 hr, respectively (*n* = 3). Both MDA-MB-231 NAT1 KO cell lines had a significant (ANOVA, *p* < 0.0001) increase in doubling time compared to the parental MDA-MB-231 cell line (Figure 4(a)).

The doubling times for the MCF-7 parental cell line and gRNA #2 and #5 NAT1 KO cell lines were 41.4 ± 0.4, 45.3 ± 6.2, and 38.8 ± 8.5 hr, respectively (*n* = 3), which did not differ significantly (ANOVA, *p* > 0.05) compared to the parental MCF-7 cell line (Figure 4(a)).

The doubling times for the ZR-75-1 parental cell line and gRNA #2 NAT1 KO cell line were 37.0 ± 2.8, and 63.1 ± 2.9 hr, respectively (*n* = 4). The doubling time of the ZR-75-1 gRNA #2 NAT1 KO cell line was significantly (Student's *t*-test, *p*=0.0006) elevated compared to the parental ZR-75-1 cell line (Figure 4(a)).

### 3.6. Anchorage-*Dependent* Colony Formation

Anchorage-*dependent* colony formation assay allows the determination of cancer cell ability to form colonies when attached to a surface. Results of the anchorage-*dependent* colony formation assay showed the number of colonies formed for MDA-MB-231 parental colonies, gRNA #2, and gRNA #5 NAT1 KO cell lines was 40.7 ± 2.5, 51.3 ± 1.6, and 54.8 ± 6.8 colonies, respectively. Anchorage-*dependent* colonies among the MDA-MB-231 and NAT1 KO cell lines did not differ statistically from each other (ANOVA, *p* > 0.05; *n* = 6) (Figure 4(b)).

MCF-7 parental and gRNA #2 and #5 NAT1 KO cell line anchorage-*dependent* colony formation was 68.2 ± 8.6, 86.2 ± 9.9, and 96.7 ± 9.1 colonies, respectively, which did not differ statistically from each other (ANOVA, *p* > 0.05; *n* = 3) (Figure 4(b)).

ZR-75-1 parental and gRNA #2 NAT1 KO cell anchorage-*dependent* colony formation was 102 ± 5, and 39.4 ± 6.5 colonies, which differed statistically from each other (Student's *t*-test, *p* < 0.001; *n* = 3) (Figure 4(b)).

### 3.7. Anchorage-*Independent* Colony Formation

Anchorage-*independent* colony formation assays (also known as “soft agar assays”) allows the determination of cancer cell ability to form colonies in the absence of cellular attachment to a surface. The MDA-MB-231 parental cell line formed anchorage-*independent* colonies at markedly higher levels than the NAT1 KO clones (Figure 4(c)). The number of colonies formed by MDA-MB-231 parental and gRNA #2 and #5 NAT1 KO cell lines were 1070 ± 76, 48.3 ± 17.2, and 23.4 ± 7.0 colonies, respectively (ANOVA, *p* < 0.0001; *n* = 3). The number of colonies formed by the two NAT1 KO cell lines were not statistically different from each other (*p* > 0.05).

The MCF-7 parental cell line formed anchorage-*independent* colonies at a higher level than the MCF-7 NAT1 KO clones (Figure 4(c)). The number of colonies formed by MCF-7 parental and gRNA #2 and #5 NAT1 KO cell lines were 195 ± 9, 33.7 ± 6.8, and 13.8 ± 6.6, respectively. Anchorage-*independent* colonies formed by the MCF-7 parental cell line were significantly higher than gRNA #2 and #5 NAT1 KO clones (ANOVA *p* < 0.0001; *n* = 4). The MCF-7 gRNA #2 and #5 NAT1 KO clones were not statistically (*p* > 0.05) different from each other.

The ZR-75-1 parental cell line formed anchorage-*independent* colonies at a higher level than the NAT1 KO cell line clone (Figure 4(c)). The number of colonies formed by ZR-75-1 parental and gRNA #2 NAT1 KO cell line was 45.6 ± 13.4, and 6.00 ± 1.84, respectively. Anchorage-*independent* colonies formed by the ZR-75-1 parental cell line were significantly higher than gRNA #2 NAT1 KO clone (Student's *t*-test, *p* < 0.05; *n* = 3).

## 4. Discussion

We investigated the effect of NAT1 gene deletion in three different human breast cancer cell lines, MDA-MB-231, MCF-7, and ZR-75-1. Human NAT1 was knocked out using CRISPR/Cas9 technology and two different guide RNAs. None of the NAT1 KO cell lines exhibited detectable NAT1 activity when measured using their selective substrate PABA. Endogenous AcCoA levels (cofactor for acetylation pathways) in NAT1 KO cell lines were significantly elevated in the MDA-MB-231 (*p* < 0.001) and MCF-7 (*p*=0.0127) but not the ZR-75-1 (*p* > 0.05). Although the effects of NAT1 KO on cell doubling time were inconsistent across the three breast cancer cell lines, the ability of the NAT1 KO cell lines to form anchorage-independent colonies in soft agar was dramatically and consistently reduced in each of the breast cancer cell lines. The NAT1 KO clones for MDA-MB-231, MCF-7, and ZR-75-1 had a reduction greater than 20-, 6-, and 7-folds in anchorage-independent cell growth, respectively, compared to their parental cell lines (*p* < 0.0001, *p* < 0.0001 and *p* < 0.05, respectively).

CRISPR/Cas9 was used to make stable NAT1 KO human MDA-MB-231, MCF-7, and ZR-75-1 breast cancer cell lines. We used two different gRNA's to allow us to distinguish between specific NAT1 KO effects versus off-target effects caused by gRNA binding and mutating at nonspecific site (s). We also used a MDA-MB-231 breast cancer cell line with a single FRT site and a nonspecific scrambled shRNA inserted in the FRT site as the MDA-MB-231 parental cell line. This cell line was transfected with gRNA #2 and #5 to facilitate comparison of the results of NAT1 KO with those previously described for NAT1 knockdown [16].

We isolated single clones from MDA-MB-231 and MCF-7 cells lines with both gRNA #2 and #5. We were not able to isolate a NAT1 KO clone using gRNA #5 in the ZR-75-1 cell line, likely due to reduced growth rate of ZR-75-1 NAT1 KO cell lines. Other groups have also investigated NAT1 KO in MDA-MB-231 and other cell lines by CRISPR/Cas9 [21, 22]. The knockout strategy of the previous studies was different from ours in the following regards: (1) different gRNA sequences were employed to cause DNA breaks and (2) previous studies used a linear donor plasmid carrying a selection marker stably integrated into the DNA breakage site. We chose transient transfection to minimize off-target effects, so the gRNA/Cas9 plasmid was present in the cell for a short time (72–96 hr). We also attempted to assess the effects of NAT1 rescue of gRNA #2 and #5 NAT1 KO in MDA-MB-231 and MCF-7 cells. Although we initially measured PABA NAT1 activity confirming successful NAT1 rescue, the NAT1 activity was no longer detectable during experiments to measure AcCoA levels or cell growth properties, and thus we were not able to characterize the effects of NAT1 rescue.

NAT1 KO in MDA-MB-231, MCF-7, and ZR-75-1 reduced levels of PABA *N*-acetylation below limits of detection both *in vitro* and *in situ*. Despite this functional validation of the NAT1 KO, the effects on endogenous levels of AcCoA and cancer cell growth were not completely consistent across different cell lines. The KO of NAT1 activity by both gRNA #2 and gRNA #5 in MDA-MB-231 breast cancer cells caused a modest but significant (*p* < 0.0001) elevation in doubling time but neither gRNA #2 or gRNA #5 caused significant (*p* > 0.05) elevation in doubling time for the MCF-7 breast cancer cell line. NAT1 KO in ZR-75-1 cells using gRNA #2 resulted in a 1.7-fold (*p* < 0.001) elevation in doubling time. Previous studies in our laboratory found that knockdown of NAT1 in MDA-MB-231 by approximately 40% did not significantly change the doubling time [16]. Knockdown of NAT1 by 85% in HT-29 cells showed similar exponential growth; however, the NAT1 knockdown cells reached saturation density earlier than control cells [15]. With NAT1 KO cells, cell death increased when cells were at confluence [15]. Wang et al. demonstrated that growth in low glucose (1 mM) was enhanced in HT29 cells following NAT1 KO [21]. We performed experiments with both MDA-MB-231 and ZR-75-1 parental and NAT1 KO cells in the presence of low (1 g/L or 5.5 mM) or no glucose supplemented with 10 mM galactose to determine whether NAT1 KO would alter the cell doubling time under these nutrient conditions. Cells grown in the presence of low (1 g/L or 5.5 mM) or no glucose supplemented with 10 mM galactose grew more slowly; however, the relationship between the parental and NAT1 KO cell lines did not differ from cells grown in standard media.

The level of NAT1 was substantially and significantly (*p* < 0.0001) reduced in each of the NAT1 KO cells lines. In MDA-MB-231 and MCF-7 cells, NAT1 KO was associated with a significant increase (*p* < 0.0001) in NAT2 protein. However, NAT2 enzymatic activity in the parental or NAT1 KO cell lines was below the limit of detection.

Human NAT1 has the capacity to hydrolyze AcCoA [3, 4] and partial knockdown of NAT1 in MDA-MB-231 cells has been reported to increase endogenous AcCoA levels [16]. In the present study, we measured the endogenous level of AcCoA in MDA-MB-231, MCF-7, and ZR-75-1 parental and NAT1 KO cell lines. The MDA-MB-231 and MCF-7 NAT1 KO with both the gRNA #2 and gRNA #5 showed significant (*p* < 0.05) increases in AcCoA levels relative to their respective parental cell lines. However, the ZR-75-1 NAT1 KO cell line did not show the same increase in AcCoA compared to the parental cell line as did the MDA-MB-231 and MCF-7 cell lines. The results for MDA-MB-231 and MCF-7 NAT1 KO cell lines are similar to what was observed when NAT1 was knocked down by shRNA in MDA-MB-231 cells [16].

AcCoA is considered a central metabolic intermediate whose level reflects the general energetic state of the cell [23]. In addition, AcCoA concentrations not only influence the activity or specificity of multiple enzymes but also influence the acetylation profiles of proteins, including histones. For instance, it is well known that the *N*^*ε*^ amino group of lysine residues can be posttranslationally modified via acetylation, the process by which numerous key cellular processes, including energy metabolism, mitosis, and autophagy, are known to be regulated [23, 24]. Notably, many lysine acetyltransferases have a relatively high *K*_D_ (low affinity) for AcCoA [25], and thus changes in cellular AcCoA levels likely affect their enzymatic activity and subsequently the acetylation profile of their substrate proteins. Interestingly, in two of the cell lines tested in the current study, NAT1 deficiency led to a significant increase in the cellular level of AcCoA, which suggests that cellular level of AcCoA is, at least in part, dependent on NAT1 activity in these cell lines. In support of this, we also have previously reported that rat embryonic fibroblasts from rapid acetylator congenic rats (high levels of rat NAT2 which is orthologous to human NAT1) have lower levels of AcCoA than those derived from slow acetylator congenic rats, which have low levels of rat Nat2 [12]. NAT1 uses AcCoA during *N*-acetylation of endogenous and exogenous substrates, but also catalyzes the hydrolysis of AcCoA to CoA in the presence of folate [3, 4]. It is possible that the cellular levels of AcCoA are negatively affected by NAT1 activity. Based on this, we can speculate that depletion of NAT1 could in turn lead to increased levels of AcCoA, which occurred in two of the three cancer cell lines investigated in our study. Depletion of NAT1 in ZR-75-1 cells did not result in an increase in the AcCoA level despite the fact that they exhibit the highest NAT1 activity among the three breast cancer cell lines tested. Alternatively, the elevated levels of AcCoA in two KO cell lines (i.e., MDA-MB-231 and MCF-7) may not be a direct effect of NAT1 depletion but rather reflect changes in their metabolic status. Whether or not elevated levels of AcCoA in NAT1 KO cell lines translate into alterations in protein acetylation profile within these cells remains unknown. Furthermore, the significant reductions in cell growth rate as well as anchorage-dependent and anchorage-independent growth observed in NAT1 KO ZR-75-1 cells occurred in the absence of concomitant changes in AcCoA levels. Thus, the relationship of AcCoA levels to alterations in cancer growth properties observed in NAT1 KO cells requires further investigation.

Anchorage-dependent colony formation in human MDA-MB-231 and MCF-7 did not show a significant difference (*p* > 0.05) between parental and NAT1 KO cell lines. These results agree with previous studies where knockdown of NAT1 in MDA-MB-231 cells by shRNA did not alter the ability of the cells to form anchorage-dependent colonies [16]. Although NAT1 KO in the ZR-75-1 cell line formed fewer colonies than the parental ZR-75-1, this difference may be due to the fact that the ZR-75-1 KO cells grew more slowly than the ZR-75-1 parental cell line.

The ability of the NAT1 KO cell lines to form anchorage-independent colonies in soft agar was dramatically and consistently reduced in each of the MDA-MB-231, MCF-7, and ZR-75-1 breast cancer cell lines. This data is consistent with previous results following knockdown of NAT1 by shRNA in MDA-MB-231 [16] and HT-29 [14, 15] cells. Although the significant decline in the ability of ZR-75-1 NAT1 KO cells to form anchorage-independent colonies may be partially attributed to its slower growth rate (i.e., a higher cell doubling time; Figure 4(a)), the magnitude of the decline (from 45.6 ± 13.4 colonies in parental cells to 6.00 ± 1.84 colonies in NAT1 KO cells; an approximately 7.6-fold decrease) was greater than that observed with anchorage-dependent colony formation (from 102 ± 5 colonies in parental cells to 39.4 ± 6.5 colonies in NAT1 KO cells; an approximately 2.6-fold decrease). Based on this, it seems that the ability of ZR-75-1 cells to form colonies in an anchorage-independent manner is further compromised in the absence of NAT1.

Anchorage-independent growth is one of the hallmarks of metastatic tumors. Tumor cells often lose epithelial features and acquire mesenchymal properties via a complex and dynamic EMT. Through EMT, the tumor cells are believed to acquire increased motility and resistance to apoptosis, ultimately leading to metastasis [26]. Savci-Heijink and colleagues analyzed gene expression signatures specifically associated with the development of bone metastases of breast cancer using primary breast tumor samples and reported NAT1 as one of three genes whose increased expression levels were highly correlated to EMT-activated breast tumor [27]. In a follow-up study, they also demonstrated a high correlation between positive immunostaining for NAT1 and expression of EMT signature genes in breast cancer [10], suggesting that increased NAT1 expression may contribute to the EMT of breast cancers and subsequently their metastatic potential. In support of this notion, we found that NAT1 KO reduced anchorage-independent growth in all three breast cancer cell lines tested. Similarly, Tiang et al. have previously reported that RNAi-mediated knockdown of NAT1 in the colon adenocarcinoma cell line, HT-29, leads to increased growth inhibition by cell-cell contact and attenuation of anchorage-independent growth in soft agar [15]. In a later study, the same group silenced NAT1 in the triple-negative breast cancer cell lines and tested the invasiveness of the cells in both *in vitro* and *in vivo*. Importantly, NAT1 knockdown in MDA-MB-231 cells resulted in a significant reduction in their ability to metastasize to and colonize in the lungs when injected into nude mice [14], suggesting that increased NAT1 level in breast cancer cells can contribute to their metastatic properties *in vivo*.

In conclusion, we knocked out human NAT1 with CRISPR/Cas9 technology using two different gRNA's in three different breast cancer cells lines. We verified complete NAT1 KO by measurement of PABA *N*-acetylation *in vitro* and *in situ* and measurement of NAT1-specific immunoreactive protein. KO of NAT1 caused a significant decrease in cell growth for MDA-MB-231 and ZR-75-1, but not for MCF-7 NAT1 KO cells relative to their respective parental cell lines. NAT1 KO caused a significant increase in cellular AcCoA levels in MDA-MB-231 and MCF-7 cells but not for ZR-75-1 cells relative to the parental cell lines. Each NAT1 KO cell line showed a dramatic decrease in the number of colonies that formed in an anchorage-independent manner relative to their respective parental cell line. Although it appears that NAT1 KO can influence the cell morphology and cell-cell interactions in cancer cell lines, further investigation is needed into whether or not NAT1 depletion ultimately alters metastatic potential *in vivo*.

## Figures and Tables

**Table 1:** Genomic DNA sequences of the reference (*NAT1*^*∗*^*4*) and mutated *NAT1* from each NAT1 KO clone.

Cell line	KO clone	No. of bp from start codon	Genomic sequence	No. of bp from start codon
	Reference (*NAT1*^*∗*^*4*)		1 ATGGACATTGAAGCATATCTTGAAAGAATTGGCTATAAGA	
			41 AGTCTAGGAACAAATTGGACTTGGAAACATTAACTGACAT	
			81 TCTTCAACACCAGATCCGAGCTGTTCCCTTTGAGAACCTT	
			121 AACATCCATTGTGGGGATGCCATGGACTTAGGCTTAGAGG	
			161 CCATTTTTGATCAAGTTGTGAGAAGAAATCGGGGTGGATG	
			201 GTGTCTCCAGGTCAATCATCTTCTGTACTGGGCTCTGACC	
			241 ACTATTGGTTTTGAGACCACGATGTTGGGAGGGTATGTTT	
			281 ACAGCACTCCAGCCAAAAAATACAGCACTGGCATGATTCA	
			321 CCTTCTCCTGCAGGTGACCATTGATGGCAGGAACTACATT	
			361 GTCGATGCTGGGTTTGGACGCTCATACCAGATGTGGCAGC	
			401 CTCTGGAGTTAATTTCTGGGAAGGATCAGCCTCAGGTGCC	
			441 TTGTGTCTTCCGTTTGACGGAAGAGAATGGATTCTGGTAT	
			481 CTAGACCAAATCAGAAGGGAACAGTACATTCCAAATGAAG	
			521 AATTTCTTCATTCTGATCTCCTAGAAGACAGCAAATACCG	
			561 AAAAATCTACTCCTTTACTCTTAAGCCTCGAACAATTGAA	
			601 GATTTTGAGTCTATGAATACATACCTGCAGACATCTCCAT	
			641 CATCTGTGTTTACTAGTAAATCATTTTGTTCCTTGCAGAC	
			681 CCCAGATGGGGTTCACTGTTTGGTGGGCTTCACCCTCACC	
			721 CATAGGAGATTCAATTATAAGGACAATACAGATCTAATAG	
			761 AGTTCAAGACTCTGAGTGAGGAAGAAATAGAAAAAGTGCT	
			801 GAAAAATATATTTAATATTTCCTTGCAGAGAAAGCTTGTG	
			841 CCCAAACATGGTGATAGATTTTTTACTATTTAG	
MDA-MB-231	gRNA #2	76	GACATTCTTCAACACCAGATC-GAGCTGTT	105
MDA-MB-231	gRNA #5	21	TGAAAGAATTGGCTATAAGAAG--TAGGAA	50
MCF-7	gRNA #2	91	CAGA-----------------------------TGTGGG	135
MCF-7	gRNA #5	31	GGCTATAAGAA-TCTAGGAACAAATTGGAC	60
MCF-7	gRNA #5	31	GGCTATAAGAAGA-----AACAAATTGGAC	60
ZR-75-1	gRNA #2	76	GACATTCTTCAACACCAGAT**A**CCGAGCTGTT (mutated nucleotide in bold face font)	105

**Table 2:** Amino acid sequences of reference (NAT1 4) and mutated NAT1 from each NAT1 KO clone.

Cell line	KO clone	Amino acid sequence	No. of total amino acids
	NAT1 4 (Reference)	MDIEAYLERIGYKKSRNKLDLETLTDILQHQIRAVPFENLNIHCGDAMDLGLEAIFDQVVRRNRGGWCLQVNHLLYWALTTIGFETTMLGGYVYSTPAKKYSTGMIHLLLQVTIDGRNYIVDAGFGRSYQMWQPLELISGKDQPQVPCVFRLTEENGFWYLDQIRREQYIPNEEFLHSDLLEDSKYRKIYSFTLKPRTIEDFESMNTYLQTSPSSVFTSKSFCSLQTPDGVHCLVGFTLTHRRFNYKDNTDLIEFKTLSEEEIEKVLKNIFNISLQRKLVPKHGDRFFTI stop	290
MB-MDA-231	gRNA #2 clone	MDIEAYLERIGYKKSRNKLDLETLTDILQHQIELFPLRTLTSIVGMPWT stop	49
MB-MDA-231	gRNA #5 clone	MDIEAYLERIGYKK stop	14
MCF-7	gRNA #2 clone	MDIEAYLERIGYKKSRNKLDLETLTDILQHQIVGMPWT stop	38
MCF-7	gRNA #5 clone (allele 1)	MDIEAYLERIGYKNLGTNWTWKH stop	23
MCF-7	gRNA #5 clone (allele 2)	MDIEAYLERIGYKKKQIGLGNIN stop	23
ZR-75-1	gRNA #2 clone	MDIEAYLERIGYKKSRNKLDLETLTDILQHQIPSCSL stop	37

## Data Availability

The data that support the findings of this study are available from the corresponding author upon reasonable request.

## Abbreviations

AcCoA: Acetyl-Coenzyme A
NAT1: *N*-acetyltransferase 1
NAT2: *N*-acetyltransferase 2
PABA: *para*-aminobenzoic acid
EMT: Epithelial-to-mesenchymal transition
gRNA: Guide RNA
KO: Knockout
PBS: Phosphate-buffered saline
EDTA: Ethylenediaminetetraacetic acid
DMEM: Dulbecco's modified eagles medium
RFU: Relative fluorescence units
HPLC: High-performance liquid chromatography.
